# Fabrication of Enzyme-Free and Rapid Electrochemical Detection of Glucose Sensor Based on ZnO Rod and Ru Doped Carbon Nitride Modified Gold Transducer

**DOI:** 10.3390/nano12101778

**Published:** 2022-05-23

**Authors:** Habibulla Imran, Asrar Alam, Venkataraman Dharuman, Sooman Lim

**Affiliations:** 1 Department of Flexible and Printable Electronics, LANL-JBNU Engineering Institute, Jeonbuk National University, Jeonju 54896, Korea; imranh091@gmail.com (H.I.); asrarlm0@gmail.com (A.A.); 2 Molecular Electronics Laboratory, Department of Bioelectronics and Biosensors, Science Campus, Alagappa University, Karaikudi 630004, India

**Keywords:** human blood, low cost, enzyme-free, glucose, ultra-sensitive, diabetes

## Abstract

Over 3 in 4 adults with diabetes live in low- and middle-income counties and health expenditure also increased 316% over the last 15 years. In this regard, we fabricate low cost, reusable and rapid detection of diabetes sensor based on zinc oxide rod inserted ruthenium-doped carbon nitride (ZnO–g–Ru–C_3_N_4_) modified sensor device. Developed sensor device physically and electrochemically characterized using X-ray diffraction (XRD), fourier-transform infrared spectroscopy (FTIR), transmission electron microscopy (TEM), X-ray photoelectron spectroscopy (XPS), cyclic voltammetry (CV), chronoamperometry (CA) and differential pulse voltammetry (DPV). Sensing device as an effective enzyme-free glucose detection with high sensitivity (346 μA/mM/cm^2^) over the applied lower potential of +0.26 V (vs. Ag/AgCl), fast response (3 s) and broad linear range of (2–28) mM, coupled with a lower limit of detection (3.5 nM). The biosensing device gives better anti-interference ability with justifiable reproducibility, reusability (single electrode re-use 26 times in physiological buffer and 3 times in serum) and stability. Moreover, the real-time applicability of the sensor device was evaluated in human blood, serum and urine samples.

## 1. Introduction

In 2021, worldwide, 537 million people had diabetes [1], which is one of the leading causes of death (in 2021, approximately 6.7 million deaths), compared to HIV/AIDS, tuberculosis, malaria and disability. In India, about 50.9 million people suffer from diabetes, and this ratio is likely to increase to 80 million by 2025, making it the ‘Diabetes Capital’ of the world. In 2021, diabetes-related health expenditure was nearly US$966 billion [1]. Since patients with diabetes have high blood glucose, accurate determination and regular checking of blood glucose level are very important in the diagnosis and treatment of diabetes or metabolic disorders and help to reduce the death rate and economic cost. To date, different methods have been reported for the detection of glucose, such as optical [2], fluorescence [3], surface plasmon resonance [4], acoustic [5] and electrochemical methods [6,7,8]. Among the various detection methods, electrochemical methods have attracted much attention because of their rapid response, ease of operation, high sensitivity and excellent selectivity [6,7,8]. In general, two types of amperometric glucose sensors have been reported. One uses glucose oxidase (GOX) enzyme to catalyze a glucose sensor. Recently, Zhang et al. reported the GOX enzyme immobilized on CNTs-based glucose sensor [9]. The other is the direct enzyme-free electrochemical oxidation glucose sensor. Baghayeri et al. reported the Ag nanoparticles functionalized CNTs-based enzyme-free glucose sensor [10]. Between them, GOX enzyme immobilization chemistry is very difficult, and is easily affected by temperature and pH. By comparison, the enzyme-free glucose sensor is simple, low cost and environmental free conditions. From the literature, carbon nanostructures, such as carbon nanotubes (CNTs), graphene and graphene oxides have been used to fabricate sensors based on glucose oxidase enzymes [9]. However, the highly expensive instruments and toxic chemical and safety issues in the synthesis of graphene, graphene oxide and CNTs mean that they have limited commercial applications. Very recently, most research has used as an alternative graphitic carbon nitride (g–C_3_N_4_). This is a 2D material and conjugated polymer that has attracted wide interdisciplinary attention as a low-cost, metal-free material with excellent electronic band structures, electron-rich properties, basic surface functions and high physicochemical stability. It has been applied in several fields, such as photosynthesis [11], H_2_ evolution [12], supercapacitor [13] and sensor [7,8]. Recently, Wang et al. developed a ternary heterostructure that consists of Ru species and C–N was rationally explored for ammonia photosynthesis [14]. Chen et al. prepared graphitic C_3_N_4_ modified by Ru (II)-based dyes for photocatalytic H_2_ evolution [15]. Shcherban et al. reported botulin oxidation over Ru nanoparticle supported on carbon nitride [16]. Peng et al. prepared Ru ion complexed carbon nitride nanosheet supported rGO as catalysts for electrochemical hydrogen evolution [17]. Recently, Hao reported a green synthesis of ruthenium modified graphitic carbon nitride nanosheet for enhanced photocatalytic ammonia synthesis [18]. However, Ru-doped g–C_3_N_4_ has not been attempted in the literature for glucose biosensing.

Glucose oxidase (GOX) enzyme and g–C_3_N_4_ modified glassy carbon electrode to detect glucose sensing with a detection limit of 11 μM was reported [19]. g–C_3_N_4_/αFe_2_O_3_ [20] and Cu^2+^–C_3_N_4_ [21] modified electrodes used to detect enzyme-free glucose sensor in KOH and NaOH medium were reported, with limits of detection of 0.58 and 0.35 µM, respectively. More recently, we have reported a niobium-doped carbon nitride [8] and platinum and zinc oxide modified with carbon nitride [7] for enzyme-free glucose detection in physiological buffer and obtained limits of detection of 1 and 0.1 µM, respectively. The limit of detection, linear range and selectivity limit their commercialization. To address this challenge, we have developed a simple, fast response, highly sensitive and selective enzyme-free glucose sensor at zinc oxide rod inserted ruthenium-doped carbon nitride sheet modified sensor device.

In this paper, we prepared a one-pot synthesis of ruthenium-doped graphitic carbon nitride (g–Ru–C_3_N_4_) sheet by an easy pyrolysis method using urea and ruthenium (III) chloride. Further, Zinc oxide rod was synthesized by precipitation method, and inserted into Ru-doped graphitic carbon nitride (ZnO–g–Ru–C_3_N_4_), prepared using a simple ultrasonication method. The structural and morphological properties of the sensor device were characterized by TEM, XRD, FTIR and XPS, and applied for enzyme-free glucose sensing. We tested the newly developed sensor device in human blood, serum and human urine sample, and found the developed sensor device to be highly suitable for practical and commercial application.

## 2. Experimental Sections

### 2.1. Materials and Method

Urea, ruthenium (III) chloride, zinc chloride, sodium chloride, potassium ferrocyanide, potassium ferricyanide, sodium phosphate monobasic and dibasic, D–glucose, biotin, riboflavin, fructose and galactose where all purchased from Sigma–Aldrich, St. Louis, MO, USA. Phosphate buffer solution (PBS, pH 7.4) containing 810 mL Na_2_HPO_4_ (100 mM) and 190 mL NaH_2_PO_4_ (100 mM) was used as electrolyte for all electrochemical measurements. The cyclic voltammetry (CV), electrochemical impedance spectroscopy (EIS), chronoamperometry (CA) and differential pulse voltammetry (DPV) were carried out using the SP-150 model Bio-Logic workstation (Seyssinet-Pariset, France, driven by EC-lab 11.25 software). A conventional three-electrode system was used, consisting of a gold electrode (3 mm diameter and 65 mm length) coated with synthesized materials as a working electrode, a platinum wire (0.5 mm diameter and 5 cm length) as counter and Ag/AgCl as a reference electrode. The chemical structure and morphology of the newly developed ZnO–g–Ru–C_3_N_4_ composite materials were confirmed by X-ray diffraction (XRD, PANalytical B.V., Lelyweg 1, Almelo, The Netherlands), Energy-dispersive X-ray spectroscopy (EDS or EDX, LEO 1530 field emission from Hitachi, Japan), Fourier-transform infrared spectroscopy (FTIR, Perkin Elmer Nicolet IS10, Waltham, MA, USA), Transmission Electron Microscopy (TEM, Hitachi H–7650 electron microscopy, Tokyo, Japan) and X-ray photoelectron spectroscopy (XPS, Theta Probe AR–XPS System, Thermo Fisher Scientific UK LTD, East Grinstead, UK). The TEM and XPS instruments available at the Jeonju center KBSI, Jeonbuk National University, Republic of Korea were employed.

### 2.2. One-Pot Synthesis of Ruthenium-Doped Graphitic Carbon Nitride (g–Ru–C_3_N_4_)

One-pot synthesis of ruthenium-doped graphitic carbon nitride (g–Ru–C_3_N_4_) by the pyrolysis method was performed using urea and ruthenium (III) chloride (RuCl_3_). Initially 20 g urea was melted, 0.1 g RuCl_3_ was added, and the solution stirred well to achieve uniform dispersion. The solution was then poured into a quartz crucible and cooled to room temperature. A single crystal-like structure was formed, which was annealed at 350 °C in a tubular furnace. After the pyrolysis process, the product was washed successively with 0.1 M nitric acid, water and acetone to remove impurities and dried in air.

### 2.3. Sensor Device Fabrication

Five mg of the obtained ruthenium-doped graphitic carbon nitride (g–Ru–C_3_N_4_) material and 10 mg of zinc oxide rod (ZnO synthesis was followed by previous works [7]) were dispersed in 1 mL double distilled water and sonicated for 15 min. The homogeneous dispersion of ZnO–g–Ru–C_3_N_4_ was drop cast on the surface of Au electrode (2 μL) and dried in air. In addition, g–C_3_N_4_, ZnO and g–Ru–C_3_N_4_ modified Au electrodes were also prepared by the above procedure for comparison. The modified Au devices were used to study the electrochemical behavior of the surfaces and glucose oxidation.

### 2.4. Preparation of Analyte and Real Sample

To study the electrode sensing response, the analyte was prepared in phosphate buffer at the concentration of 100 mM for electrochemical measurement. Dynamic variation of analyte was prepared by serial dilution from 0.1 M prepared stock solution of the analytes.

Blood samples required for the experiments were collected from patients and analyses were performed in compliance with the ethical committee constituted by the Alagappa University and functioning as per ethical guidelines for biomedical research on human subjects issued by the Indian council of Medical Research (IEC/Au/2016/1/2), India. Informed consent was obtained for any experimentation with the respective patient. In this work, one blood and urine sample are used for the detection of glucose. That sample was collected from the first author. The blood and urine sample preparation and detection of glucose are followed by our previously reported processer [7,8].

## 3. Results and Discussion

### 3.1. Electrochemical Characterization of the ZnO–g–Ru–C_3_N_4_ Composite

**Figure 1:**
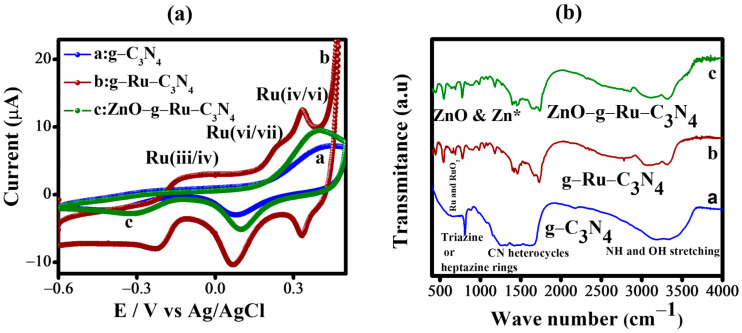
(**a**) CV behaviors of AuE-g-C_3_N_4_ (curve a), AuE-g-Ru-C_3_N_4_ (curve b) and AuE-ZnO-g-Ru-C_3_N_4_ (curve c) modified electrode in the presence of 1 M NaOH in DD water at a scan rate 50 mV s^−1^. (**b**) FT-IR spectra of g-C_3_N_4_ (curve a), g-Ru-C_3_N_4_ (curve b) and ZnO-g-Ru-C_3_N_4_ (curve c). * Skeletal vibration with carbon nitride.

To prove the electrochemical characteristics of the fabricated bare AuE, g-C_3_N_4_, g- Ru-C_3_N_4_, ZnO-g-Ru-C_3_N_4_ modified AuE, cyclic voltammetry (CV) performance acquired in 1 M NaOH were scanned between the potential of −0.6 V and 0.4 V at a scan rate of 50 mV s^−1^ is shown in Figure 1a. A CV signals of Ru-doped g-C_3_N_4_ shows three distinct redox peaks at -0.10, 0.23 and 0.34 V corresponding to Ru (III/IV), Ru (IV/VI) and Ru (VI/VII) transition processes, respectively [22]. These results clearly indicate that the newly formed g-Ru-C_3_N_4_ hybrids have newer functionalities by the doping of Ru in graphitic carbon nitride structure, which are different from pure g–C_3_N_4_ and ZnO-g-Ru-C_3_N_4_ counterparts. In contrast, RuO_2_ redox transition peaks on the ZnO-g-Ru-C_3_N_4_ are not well defined, they are mostly sluggish in nature and the peak current values are very minimum when compared to the g-C_3_N_4_ and g-Ru-C_3_N_4_ modified electrodes. We further examined the electrochemical characteristics of the various electrodes, CV and EIS performance were acquired in 1 mM [Fe(CN)_6_]^3−/4−^ as redox couple at a scan rate of 50 mV s^−1^ is shown in Figure S1. A well-resolved oxidation/reduction peak was observed in the bare AuE with an electrochemical peak separation (ΔE_p_) of 88 mV and higher peak current of 7.32 μA and R_ct_ of 2043 Ω cm^−2^. A modest increase of redox peak currents (9.58 μA) with the reduced peak separation of 80 mV and Rct of 1119 Ω cm^−2^ was obtained for g-C_3_N_4_ modified Au surface modified electrode. The increased electron transfer rate coupled with an enhanced current response and a reduced ΔE_p_ value suggest that carbon nitride contains a higher number of NH group, and it highly interacts with [Fe(CN)_6_]^3−/4−^ ions. By modification with g–Ru-C_3_N_4_, we observed slightly decrease oxidation peak current (8.58 µA) with increased ΔE_p_ (90 mV) and R_ct_ of 2217 Ω cm^−2^. Similarly, the peak current response (1.44 µA) with increased peak to peak separation (110 mV) and larger R_ct_ value (18,550 Ω cm^−2^) was observed for ZnO-g-Ru-C_3_N_4_ electrode, which might be the reason that the presence of higher amount of ZnO hampered the electron transport of [Fe(CN)_6_]^4-/3-^ due to its semi-conductive behavior.

### 3.2. Physical Characterization of ZnO–g–Ru–C_3_N_4_ Composite

Figure 1b shows the FTIR spectra of g–C_3_N_4_ (curve a), g–Ru–C_3_N_4_ (curve b) and ZnO–g–Ru–C_3_N_4_ (curve c). The g–C_3_N_4_ shows a sharp absorption peak at 810 cm^−1^ for the out-of-plane bending vibration of triazine rings. The peaks at 1262 and 1421 cm^−1^ represent the aromatic CN stretching [23]. The absorption peak at 3182 cm^−1^ is attributed to –NH and –OH stretching. In the case of the Ru-doped g–C_3_N_4_, the triazine peak at 785 cm^−1^ is shifted and the absorption peak intensity is also decreased, compared to the g–C_3_N_4_ (810 cm^−1^). In the spectrum, the small peaks at 662, 540 and 444 cm^−1^ are associated with the characteristic vibrational modes of υRu-O and γRu-O, which indicate the formation of RuO_2_ [24,25]. The peak at 849 cm^−1^ is attributed to the Ru–N stretching modes [26]. This result indicates Ru doped on the NH group of the carbon nitride structure. Further, zinc oxide is composited with g–Ru–C_3_N_4_. The 1459 and 555 cm^−1^ absorption peaks represent the skeletal vibration of g–C_3_N_4_ sheets and Zn–O stretching vibration, respectively [27]. These results indicate that ZnO binds with the g–Ru–C_3_N_4_ matrix. The g–C_3_N_4_ compared with the g–Ru–C_3_N_4_ and ZnO–g–Ru–C_3_N_4_, CN heterocycles, triazine and –NH or –OH stretching absorption peaks at 810, 1262, 1421 and 3182 cm^−1^ are shifted to 785, 1406, 1465, 1679, 3097 and 3322 cm^−1^ for g–Ru–C_3_N_4_, and to 778, 1395, 1459, 1655, 3093 and 3319 cm^−1^ for ZnO–g–Ru–C_3_N_4_, respectively. The FTIR results indicate the formation of the ZnO–g–Ru–C_3_N_4_ composite-like structure (Figure 1b).

**Figure 2:**
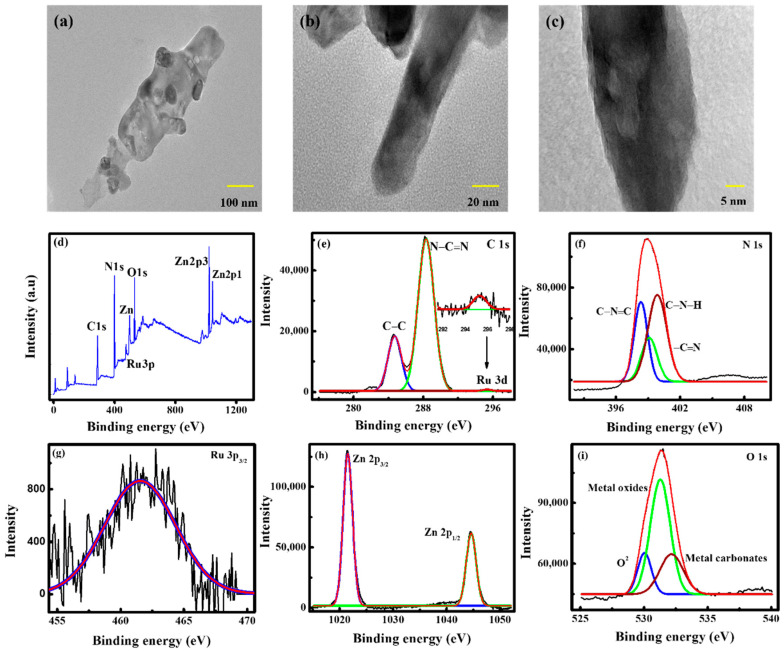
TEM images (**a**–**c**) and XPS spectrum (**d**–**i**) of ZnO–g–Ru–C_3_N_4_ composite. Surveys scan (**d**), C 1 s (**e**), N 1 s (**f**), Ru (**g**), Zn 2P (**h**) and O 1 s (**i**).

The XRD patterns in Figure S2 of the Supplementary Materials show the crystalline nature and phase planes of g–C_3_N_4_, g–Ru–C_3_N_4_ and ZnO–g–Ru–C_3_N_4_. Generally, g–C_3_N_4_ contains two peaks that are found at 2θ 13.38° and 27.38°, corresponding to the 100 and 002 planes, respectively [28]. The thermally prepared g–C_3_N_4_ shows a very weak broad peak at 27.4° corresponding to the 002 plane, suggesting the absence of long-range order in the atomic arrangements in the CN. In the presence of Ru on g–C_3_N_4_, it shows increased peak intensity at 27.4°, and no new peak appears, which indicates the Ru doped on CN matrix of the structure. Further, for the ZnO decorated on g–Ru–C_3_N_4_, the 27.4° peak still appears, but the peak intensity is very low, compared to the g–C_3_N_4_ and g–Ru–C_3_N_4_. Hence, there is a high level of ZnO loading on the Ru–CN matrix. The ZnO (JCPDS: 89–7102) peaks [7] are negatively shifted, and their intensity is increased. These results indicate the formation of composite-like structure. Figure S3 of the Supplementary Materials shows the corresponding energy dispersive X-ray (EDX) spectra of g–Ru–C_3_N_4_ and ZnO–g–Ru–C_3_N_4_, revealing the presence of carbon (C), nitrogen (N) and ruthenium (Ru), with C:N:Ru ratios of 32.67:65.81:1.52. EDX analysis confirmed that the composition of the products was Ru-doped g–C_3_N_4_, without impurities. No diffraction peaks from other metals (impurities) were found, clearly showing that the structure of the carbon nitride was not affected by Ru doping, as it is doped at the outer plane of Ru–N stretching. After the ZnO nanoparticle composite with g–Ru–C_3_N_4_, Zn (75.57 %) and O (9.79 %) elements appear, and the C:N:Ru element weight percentages decrease to 5.64 : 8.07 : 0.93, compared to ruthenium doped carbon nitride (32.67:65.81:1.52), due to the high amounts of Zn and O (75.57 + 9.79 = 85.36) attach with Ru–CN matrix. These result correlate with the XRD (Figure S2), FTIR (Figure 1b) and electrochemical data (Figure 1a). Further, the morphology of the ZnO–g–Ru–C_3_N_4_ composite was studied by transmission electron microscopy (TEM). Figure 2a–c shows the TEM imagery of ZnO–g–Ru–C_3_N_4_ at different scales of 100, 20 and 5 nm. The TEM imagery clearly indicates that the carbon nitride forms a 2D sheet-like structure, and Ru nanoparticles appear in front of the sheet, and ZnO rod is inserted on the edge of the sheet. These inserted ZnO rod enhances the sensitivity and reduces the glucose oxidation potential. The chemical composition and elements of the ZnO–g–Ru–C_3_N_4_ composite are studied by XPS, as shown in Figure 2d–i. Figure 2d shows the survey spectrum of ZnO–g–Ru–C_3_N_4_ composite revealing the C, N, Ru, Zn and O elements. However, no new element peak is shown, indicating that the composite is purely composed of only C, N, Ru, Zn and O. The C 1s XPS spectrum for ZnO–g–Ru–C_3_N_4_ (Figure 2e) displays three peaks at 284.7, 288.3 and 295.3 eV. The peaks at 284.7 and 288.3 eV represented the *sp^2^* hybridized C–C bond [29] and the *sp^2^* hybridized N–C=N carbon in the aromatic tri-s-triazine rings, respectively. However, another peak at 295.3 eV is assigned to Ru 3d. The N 1s spectrum shows three peaks centered at 398.3, 399.1 and 399.9 eV, which represent the *sp^2^* hybridized carbon–nitrogen (C–N=C) in the aromatic triazine ring, nitrogen between the aromatic rings (–C=N) and the free amino functional groups (C–N–H) of g–C_3_N_4_, respectively [30]. The Ru 3p XPS spectrum shows one absorption peak at 461.6 eV, which represents the Ru^4+^ [31]. Figure 2h shows the XPS spectrum of Zn 2p, which reveals two peaks at 1020.3 and 1044.8 eV that correspond to the Zn 2p_5/2_ and Zn 2p_3/2_, respectively. The Zn 2p spectrum indicates the presence of Zn ions with +2 oxidation states [32]. The O 1s XPS spectrum is deconvoluted into three peaks at 530, 531.2 and 532.3 eV, which are due to the O^2+^, metal oxides and metal carbonate, respectively [7]. These results clearly indicate that Zn and Ru bind with the NH matrix of the structure.

### 3.3. Enzyme-Free Glucose Sensing at ZnO–g–Ru–C_3_N_4_ Modified Sensor Device

**Figure 3:**
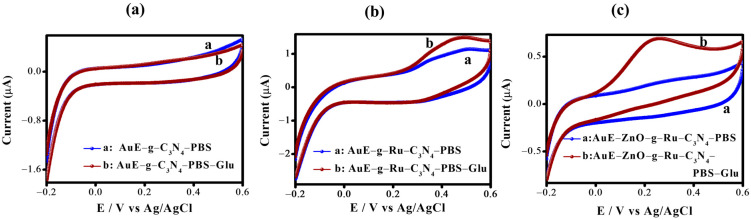
CV behavior of g–C_3_N_4_ (**a**), g–Ru–C_3_N_4_ (**b**) and ZnO–g–Ru–C_3_N_4_ (**c**) modified electrode and used for the glucose sensing. Measured in PBS in the absences (curve a) and presences (curve b) of 1 mM glucose at a scan rate 50 mV s^−1^.

**Scheme 1:**
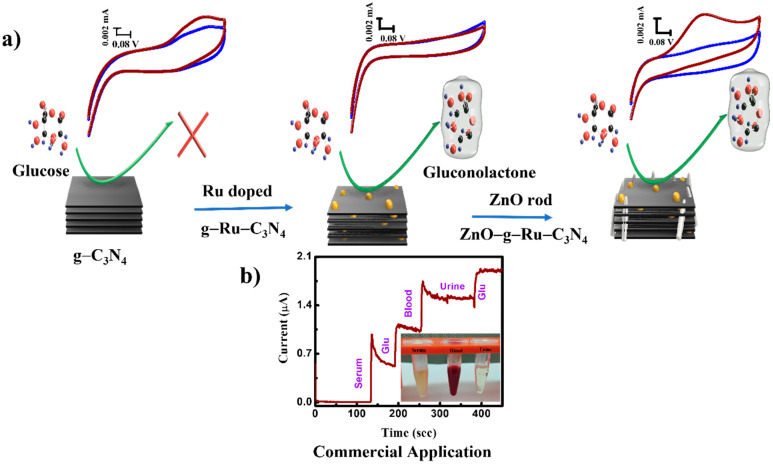
(**a**) Enzyme-free glucose sensing at ZnO rod inserted Ru doped carbon nitride modified sensor devices. (**b**) Developed sensor device practically tested in human blood, blood serum and urine samples.

The electrochemical characteristics of various modified electrodes were systematically performed towards the oxidation of glucose was evaluated by using CV at a scan rate of 50 mV s^−1^ in the absence and presence of 1 mM glucose in PBS. Interestingly, in the absence of glucose, all fabricated electrodes do not show any anodic peak current response. However, in the presence of glucose, the unmodified Au electrode (Figure S4a) peak current does not change in neutral pH, and this result corroborates previous report [33]. Similarly, Au electrode modified with g–C_3_N_4_ and ZnO was manifested only background current, which indicated an inactive electrolyte. In contrast, in the presence of glucose (1mM), a well-defined anodic peak current for glucose oxidation was obtained at 490 mV for the gold electrode modified with g–Ru–C_3_N_4_, implies that the g–Ru–C_3_N_4_ modified electrode catalyzed the glucose to gluconolactone in physiological buffer (Scheme 1, Figure 3) without aid of enzyme. Upon the modification of the gold electrode with ZnO rod decorated on Ru-doped carbon nitride (ZnO–g–Ru–C_3_N_4_), the oxidation peak current enhances remarkably (6.9 × 10^−7^ A), which is much higher than the g–Ru–C_3_N_4_ modified electrode (Figure 1). In addition, oxidation peak potential is negatively shifted (260 mV), compared to g–Ru–C_3_N_4_ (E_pa_ 490 mV) modified electrode. The outstanding electrochemical properties of the electrode are believed to be due to the synergistic interaction between g–Ru–C_3_N_4_ and ZnO nanorod, which accentuated the electrode’s electro-catalytic properties. Metal and/or metal oxide modified surface catalyzing the glucose oxidation is reported by Hsia et al. [34]. Hydroxyl ions are adsorbed on the ZnO–g–Ru–C_3_N_4_ modified surface, and act as a glucose oxidation mediator and surface oxygen reduction reaction. ZnO–g–Ru–C_3_N_4_ modified electrode catalyzes the glucose to gluconolactone, releasing two protons per glucose molecule (Scheme 1). Furthermore, the optimization of the ratio between the g–Ru–C_3_N_4_ and ZnO was studied by preparing the ZnO–g–Ru–C_3_N_4_ composites at different ratio (1:1, 1:2 and 2:1), and measured in PBS (pH 7.4) in the absence (curve a) and presence (curve b) of 1 mM glucose at a scan rate of 50 mV s^−1^ (Figure S5). Note that the ZnO–g–Ru–C_3_N_4_ composite prepared at 1:2 ratio exhibits the highest glucose oxidation compared to the other ratios (1:1 and 2:1). Thus, 1:2 ratio of g–Ru–C_3_N_4_ and ZnO is used for further electrochemical and physical characterization, and to optimize the glucose sensing properties.

### 3.4. Effect of the Scan Rate and pH on the ZnO–g–Ru–C_3_N_4_ Modified Sensor Device

**Figure 4:**
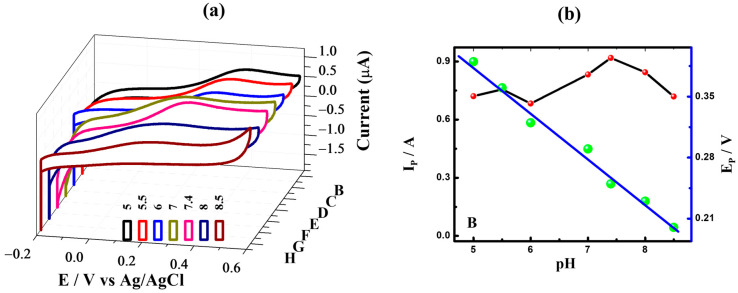
(**a**) CV behaviour of ZnO–g–Ru–C_3_N_4_ electrode in the presences of 1 mM glucose in different pHs (5 (B), 5.5 (C), 6, (D), 7 (E), 7.4 (F), 8 (G) and 8.5 (H)) solution. (**b**) pH versus peak current (left) and peak potential (right) measured from the corresponding CV behavior.

Figure S6 of the Supplementary Materials shows the effect of the scan rate varied from 10 to 100 mV s^−1^ in the presence of 1 mM glucose in PBS. From the result, the scan rate is increased and the glucose oxidation peak current also increases, due to its diffusion-controlled (1.75 × 10^−6^ cm^−1^) oxidation of glucose to gluconolactone [35,36,37,38,39,40]. Next, the effect of the pH on the ZnO–g–Ru–C_3_N_4_ modified electrode in the presences of 1 mM glucose oxidation peak current and peak potential was carefully monitored by CV using 100 mM PBS in different pH ranging (5, 5.5, 6, 7, 7.4, 8 and 8.5) at a scan rate 50 mV s^−1^ in Figure 4. It is apparent that the glucose oxidation peak current is increased to pH 7.4, then the oxidation peak current is decreased. From Figure 4, the highest glucose oxidation peak current is obtained at pH 7.4. In addition, as the PBS solution pH (5 to 8.5) was gradually increased, the glucose oxidation peak potential was positively shifted, which indicates that protons are involved in the electrode processes [41,42]. These results indicate that pH of 7.4 results in higher glucose oxidation current, and it was used for further optimization studies. The Nernst equation (ΔE_p_/(58.65 ΔpH) *= n* is used to calculate the number of electrons involved in the oxidation reaction. From slope of pH versus peak potential (E_p_), obtained 51 mV/pH for the oxidation of glucose, which indicates one electron transfer during the rate-determining step.

### 3.5. Effect of Glucose Concentration on the ZnO–g–Ru–C_3_N_4_ Modified Sensor Device

**Figure 5:**
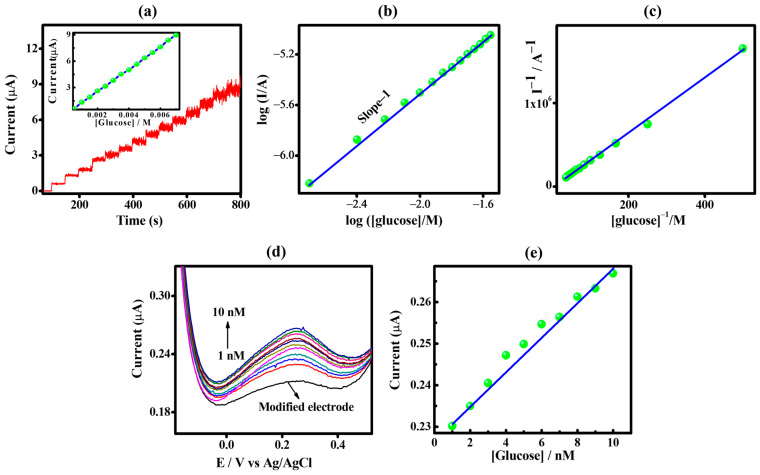
(**a**) Effect of different glucose concentration on CA behavior of ZnO–g–Ru–C_3_N_4_ measured at 0.26 V in PBS pH 7.4. Concentration range 2 to 26 mM and inset glucose concentration versus current plot. (**b**) log concentration versus log current. (**c**) Line weaver Burk plot (1/current vs. 1/ [glucose]). (**d**) Effect of glucose concentration on DPV behavior of ZnO–g–Ru–C_3_N_4_ measured in PBS pH 7.4. Concentration range 1 to 10 nM (**d**) for ZnO–g–Ru–C_3_N_4_ modified electrode and (**e**) glucose concentration versus current plot.

Figure 5a demonstrates the amperometry behavior of the newly developed ZnO–g–Ru–C_3_N_4_ modified surface with 2 mM glucose concentration sequentially added at every 50 s up to 28 mM at an applied potential of +0.26 V under the stirring condition in 100 mM PBS solution. By the successive addition of different concentration of glucose, within 3 s, the electrocatalytic oxidation current quickly increases, and the maximum signal/noise ratio 0.9 is observed. The insert images show the linear increase of glucose concentration with an increase of current and the r^2^ (0.99) values are found to be high. Figure 5b plots the log glucose concentration versus log current. From the figure, the higher glucose concentration linear ranges 2 to 28 mM with r^2^ value of 1. These newly developed ZnO–g–Ru–C_3_N_4_ modified sensor device are suitable for applying Michaelis–Menten (MM) kinetics. From the line weaver Burk plot (Figure 5c), a linear equation is obtained of I^−1^ = (8.16 × 10^3^) × [glucose]^−1^ + (1.24 × 10^3^). The K_M_ is calculated as 65 mM, which is the highest K_M_ value, compared to the g–C_3_N_4_ based systems.

Further, the limit of detection (LOD) is calculated from the difference pulse voltammetry (DPV) in Figure 5d,e. The ZnO–g–Ru–C_3_N_4_ shows good linearity, even in a low concentration range of 1 to 10 nM and a correlation coefficient of 0.99. The LOD has been theoretically calculated using the formula 3× standard deviation of the blank signal (4.71 × 10^−9^ A)/slope of the calibration curve (4.029 A/M) which gives the LOD 3.5 nM. The detection performance of the fabricated modified electrode is compared with other reported g–C_3_N_4_ based non-enzymatic glucose sensors. As shown in Table 1 [7,8,19,20,21] it is worth mentioning that the ZnO–g–Ru–C_3_N_4_ modified electrode exhibits outstanding electrocatalytic performance for glucose sensing in terms of ultra-high sensitivity, lower detection limit and wide linear range.

### 3.6. Selectivity, Stability and Reproducibility of the ZnO–g–Ru–C_3_N_4_ Modified Sensor Device

Selectivity, stability and reproducibility are important parameters for developing a new sensor device. The selectivity of the ZnO–g–Ru–C_3_N_4_ modified electrode for glucose sensing was studied by chronoamperometric (CA) detection using the same potential interferences as 0.1 mM fructose, galactose, NaCl, KCl, H_2_O_2_, uric acid, epinephrine, serotonin, biotin, bilirubin and riboflavin in 100 mM PBS that are present in Figure S7 of the Supplementary Materials. The potential interferences were added, respectively, in the same solution. These results clearly indicate that only the strong glucose oxidation peak current was observed, and very small current response was observed for other potential interferences. From Figure S7, the sensitivity of the developed sensor was 346 μA mM^−1^ cm^−2^ and in the presence of potential interference (such as uric acid) sensitivity of the sensor slightly increase to 354 μA mM^−1^ cm^−2^.

Figure S8 of the Supplementary Materials shows the 20 days (d) continuous monitoring of the ZnO–g–Ru–C_3_N_4_ modified electrode in the presence of 1 mM glucose in PBS at a scan rate 50 mV s^−1^ using the CV method. The anodic glucose oxidation peak current is plotted against the number of days. Compared to the day 1 oxidation peak current, only 0.6% loss was shown for day 20. These results indicate that the ZnO–g–Ru–C_3_N_4_ modified electrode shows high stability up to day 20. Further surface reproducibility is tested using 4 different ZnO–g–Ru–C_3_N_4_ modified electrodes in the presence of 1 mM glucose (Figure S9 of the Supplementary Materials). The 4 modified different electrodes show similar oxidation potential and peak current, which indicates that the surface is highly reproducible.

### 3.7. Reusability of the ZnO–g–Ru–C_3_N_4_ Modified Sensor Device

An enzyme-free and reusable surface is needed for low-cost glucose strip fabrication. In this regard, we have tested the reusability of electrode surface in PBS and blood serum. Figure S10 of the Supplementary Materials plots the anodic glucose oxidation current versus the number of times reusable in a single electrode. From the figure, the ZnO–g–Ru–C_3_N_4_ modified single sensor device is reusable in PBS for 26 times and in serum for 3 times. These results indicate that the ZnO–g–Ru–C_3_N_4_ modified electrode shows high sensitivity, very low limit of detection, selectivity, reproducibility, stability and reusability. In this regard, the newly developed sensor device was further tested for practical application.

### 3.8. Practical Application

The treatment of diabetic-related diseases is purely dependent upon the monitoring of glucose in human blood, blood serum and urine samples (Figure S11 of the Supplementary Materials). Hence, the commercial applicability of the newly developed ZnO–g–Ru–C_3_N_4_ modified sensor device was further tested for the detection of glucose in human blood, blood serum and urine samples by the chronoamperometric method. Blood and urine samples were collected from first author. The blood, serum and urine samples were successfully added in 100 mM PBS, and their corresponding amperometric response was noticed. In blood and serum, a good response was observed, while in the urine sample, no response was observed. This result indicates that the ZnO–g–Ru–C_3_N_4_ modified electrode selectively detects glucose only. The advantages of the newly fabricated electrode for glucose sensor are its simplicity, high sensitivity, very low limit of detection, selectivity and reusability.

## 4. Conclusions

A novel zinc oxide rod inserted Ru-doped graphitic carbon nitrate was prepared by a simple mixing method (5 mg g–Ru–C_3_N_4_ and 10 mg ZnO). In NaOH, the Ru-doped g–C_3_N_4_ showed three redox peaks of Ru(iii/iv), Ru(vi/vii) and Ru(iv/vi), which confirmed that the Ru nanoparticles were attached onto the CN matrix. After ZnO was composited on g–Ru–C_3_N_4_, these three redox peaks disappeared, which indicates the formation of composite-like structure. The chemical investigation using FTIR found 444, 540, 662, 849, 1459 and 555 cm^−1^ peaks that confirmed the Ru and ZnO on the g–C_3_N_4_ matrix. This binding may be due to the strong interaction of ZnO and Ru particles, and the N–H stretching group in the carbon nitride. The morphology of the composite was studied using TEM. Finally, the transducer was tested for enzyme-free glucose sensing by cyclic voltammetry, chronoamperometry and differential pulse voltammetry measurement, which results indicate the better linearity of 2 to 28 mM, high sensitivity (346 μA/mM/cm^2^), selectivity, very low limit of detection (3.5 nM), stability and reusability. This modified single sensor device is re-used 26 times in PBS and 3 times in blood serum. The practicability of the sensor is proved through the real-time determination of the glucose level in human blood, blood serum and urine samples. The overall results show that the ZnO–g–Ru–C_3_N_4_ modified electrode is a suitable platform for the clinical analysis of glucose.

## Figures and Tables

**Table 1:** Carbon nitride-based glucose sensing system.

Surface	Method	Electrolyte	Limit of Detection (LOD)	Reference
ZnO–g–Pt–C_3_N_4_	Electrochemical method	PBS	0.1 µM	[7]
g–Nb–C_3_N_4_	Electrochemical method	PBS	1 µM	[8]
g–C_3_N_4_–GOx/GCE	Electrochemical method	PBS	11 µM	[19]
g–C_3_N_4_/α–Fe_2_O_3_	Electrochemical method	KOH	0.58 µM	[20]
Cu^2+^–C_3_N_4_/MWCNT	Electrochemical method	NaOH	0.35 µM	[21]
ZnO–g–Ru–C_3_N_4_	Electrochemical method	PBS	3.5 nM	This work

## Data Availability

Not applicable.

## Supplementary Materials

The following supporting information can be downloaded at: https://www.mdpi.com/article/10.3390/nano12101778/s1, Figure S1: CV (A) EIS (B) behaviors of AuE (curve a), AuE–g–C_3_N_4_ (curve b), AuE–ZnO (Curve c), AuE–g–Ru–C_3_N_4_ (curve d) and AuE–ZnO–g–Ru–C_3_N_4_. Measurements are made in presence of [Fe(CN)_6_]^3–/4–^ in PBS (pH 7.4). Star line: [R(Q(RW))] circuit fit data.; Figure S2: XRD pattern (A) of g–C_3_N_4_ (curve a), g–Ru–C_3_N_4_ (curve b), and ZnO–g–Ru–C_3_N_4_ (B).; Figure S3: Energy dispersive X-ray (EDX) analyses of Ru–doped g–C_3_N_4_ (A) and ZnO–g–Ru–C_3_N_4_ (B).; Figure S4: CV behavior of unmodified gold electrode (A) and ZnO (B) modified electrode in the absences (curve a) and presences (curve b) of 1 mM glucose in PBS at a scan rate of 50 mV s^−1^.; Figure S5: Effect of different ratio of g–Ru–C_3_N_4_ and ZnO mixture (ratio 1:1 (A), 1:2 (B) and 2:1 (C)) used for AuE modification and used for the glucose sensing. Measured in PBS in the absences (curve a) and presences (curve b) of 1 mM glucose at a scan rate 50 mV s^−1^.; Figure S6: (A) Effect of varying scan rate on the CV behavior of ZnO–g–Ru–C_3_N_4_. (B) Corresponding plots of (scan rate)^1/2^ versus current.; Figure S7: Selective sensing of glucose with the potential interferences are fructose, galactose, riboflavin, billurubin, biotin, NaCl, KCl, H_2_O_2_, uric acid, serotonin and epinephrine with the concentration of 0.1 mM in PBS.; Figure S8: Study of stability of ZnO–g–Ru–C_3_N_4_ modified electrode monitored voltammetrically in presence of 1mM glucose in PBS. Anodic peak current is plotted against Number of days.; Figure S9: Reproducibility data obtained on using four different gold electrodes of similar activity for modifying with ZnO–g–Ru–C_3_N_4_. Measured in PBS in the absences (curve a) and presences (curve b) of 1 mM glucose at a scan rate of 50 mV s^−1^.; Figure S10: Reusable single electrode is measured in PBS (A) in the presences of 1 mM glucose at a scan rate of 50 mV s^−1^. (B) Blood serum.; Figure S11: Developed sensor device practically tested in human blood, blood serum and urine samples.
