# Supplementary material for: Fabrication of Enzyme-Free and Rapid Electrochemical Detection of Glucose Sensor Based on ZnO Rod and Ru Doped Carbon Nitride Modified Gold Transducer
Source: Nanomaterials (Basel). 2022 May 23;12(10):1778. doi: 10.3390/nano12101778 (PMC9143380; doi:10.3390/nano12101778)
Supplement: Supplementary file 1 [file nanomaterials-12-01778-s001.zip › nanomaterials-1705957-supplementary.pdf]

nanomaterials

Supplementary Materials

Fabrication of Enzyme-Free and Rapid Electrochemical Detection of Glucose Sensor Based on ZnO Rod and Ru Doped Carbon Nitride Modified Gold Transducer

Habibulla Imran [1,2], Asrar Alam [1], Venkataraman Dharuman [2,*] and Sooman Lim [1,*]

1 Department of Flexible and Printable Electronics, LANL-JBNU Engineering Institute, Jeonbuk National University, Jeonju 54896, Korea; imranh091@gmail.com (H.I.); asrarlm0@gmail.com (A.A.)
2 Molecular Electronics Laboratory, Department of Bioelectronics and Biosensors, Science Campus, Alagappa University, Karaikudi 630004, India
* Correspondence: dharumanudhay@yahoo.com (V.D.); smlim@jbnu.ac.kr (S.L.)

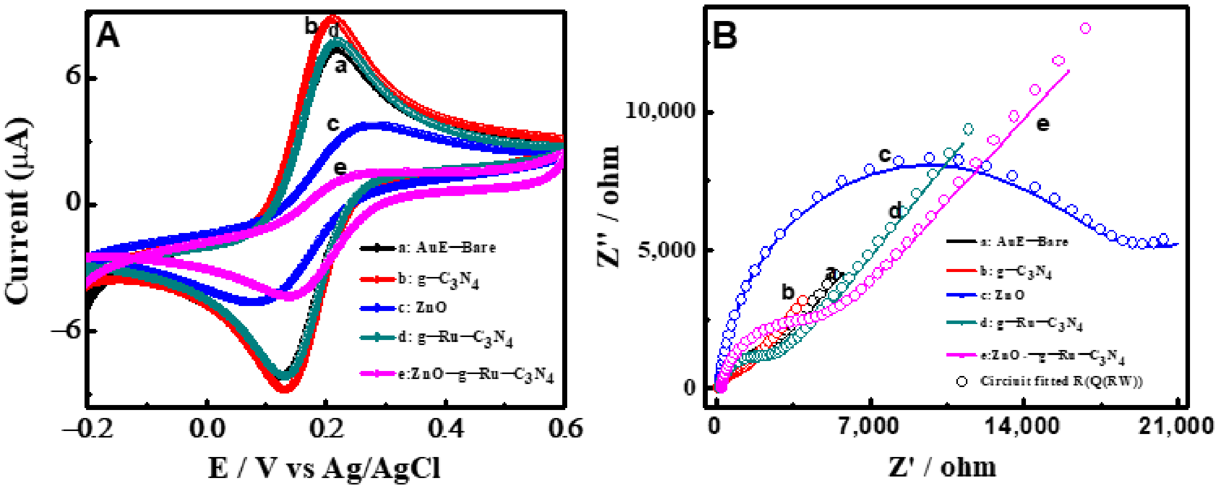

Figure S1. CV (**A**) and EIS (**B**) behaviors of AuE (curve a), AuE–g–C_3N_4(curve b), AuE–ZnO (Curve c), AuE–g–Ru–C_3N_4 (curve d) and AuE–ZnO–g–Ru–C_3N_4. Measurements are made in presence of $[Fe(CN)_6]^{3-/4-}$ in PBS (pH 7.4).Star line: [R(Q(RW))] circuit fit data.

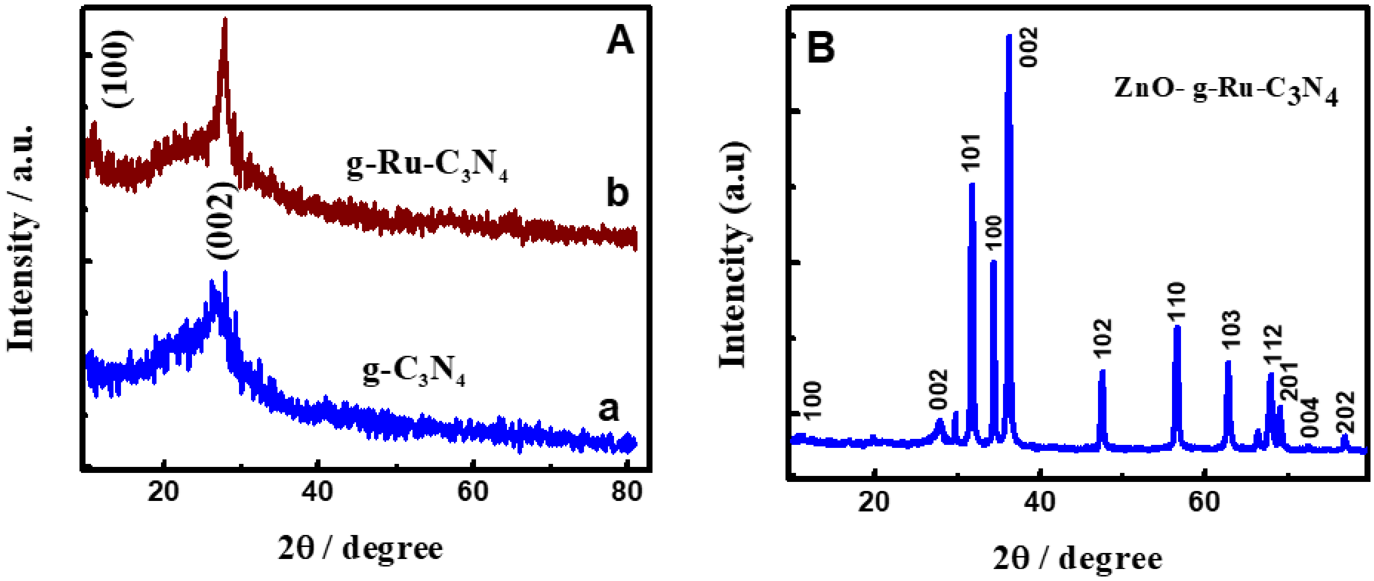

Figure S2. XRD pattern (**A**) of g-C_3N_4 (curve a), g-Ru-C_3N_4 (curve b), and ZnO–g–Ru–C_3N_4 (**B**).

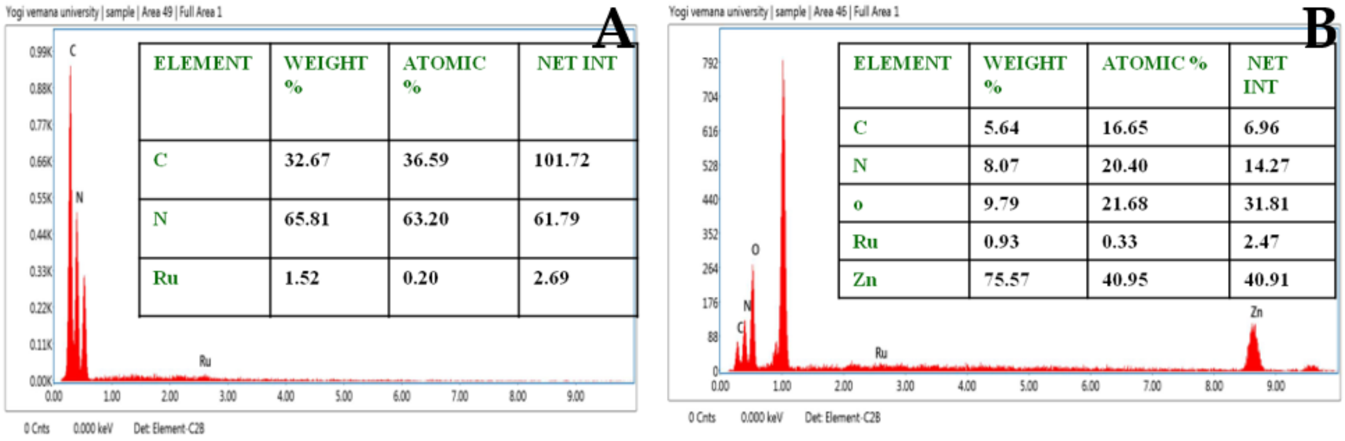

Figure S3. Energy dispersive X-ray (EDX) analyses of Ru-doped g-C_3N_4 (**A**) and ZnO–g–Ru–C_3N_4 (**B**).

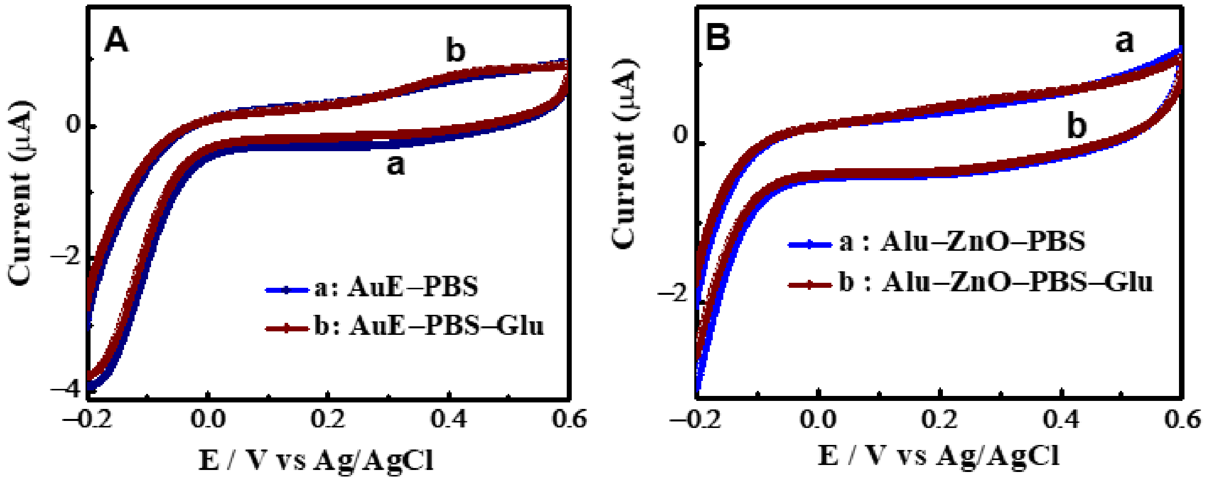

Figure S4. CV behavior of unmodified gold electrode (**A**) and ZnO (**B**) modified electrode in the absences (curve a) and presences (curve b) of 1 mM glucose in PBS at a scan rate of 50 mV s^{-1}.

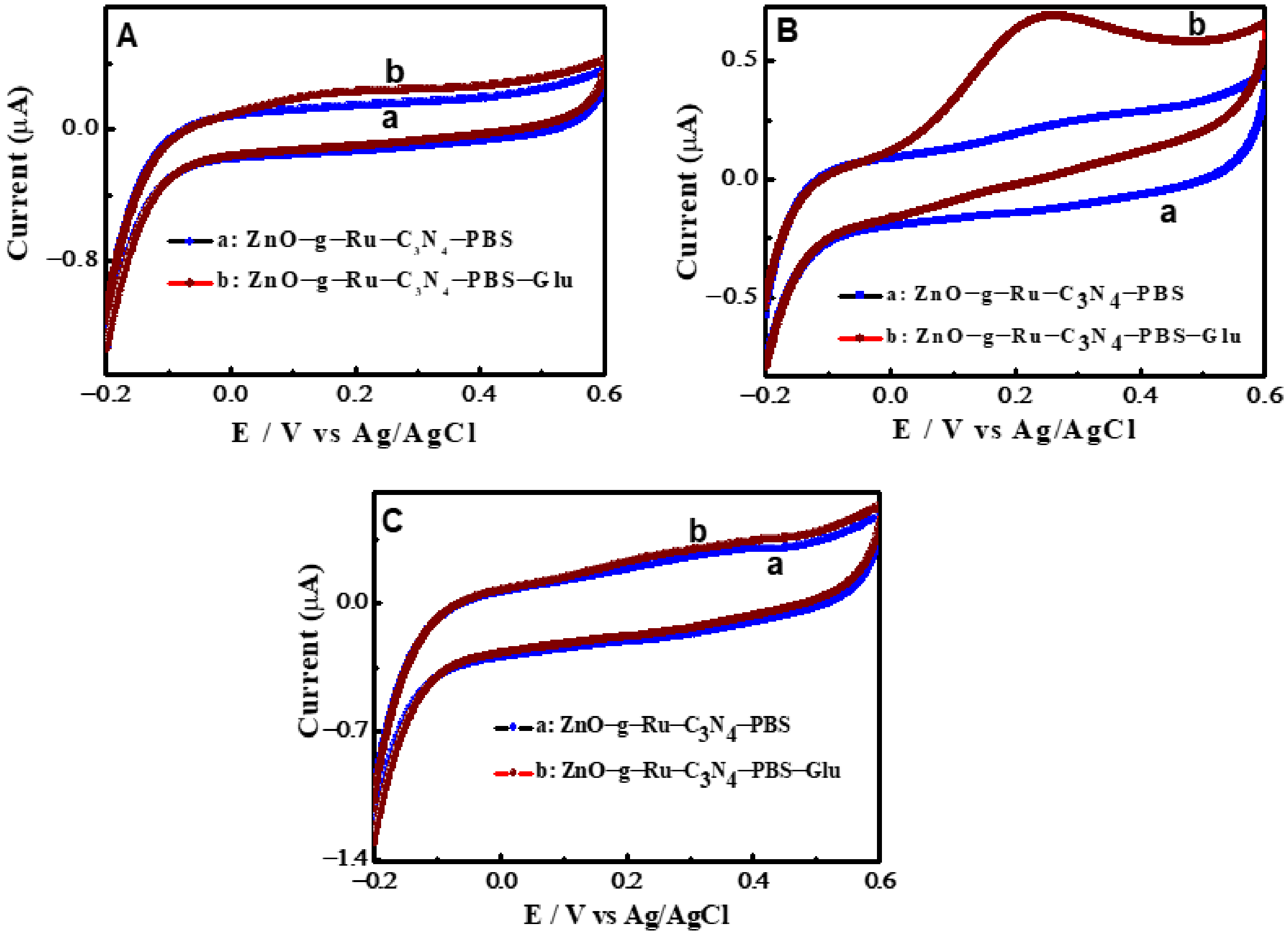

Figure S5. Effect of different ratio of g–Ru–C_3N_4 and ZnO mixture (ratio 1:1 (**A**), 1:2 (**B**) and 2:1 (**C**)) used for AuE modification and used for the glucose sensing. Measured in PBS in the absences (curve a) and presences (curve b) of 1 mM glucose at a scan rate 50 mV s^{-1}.

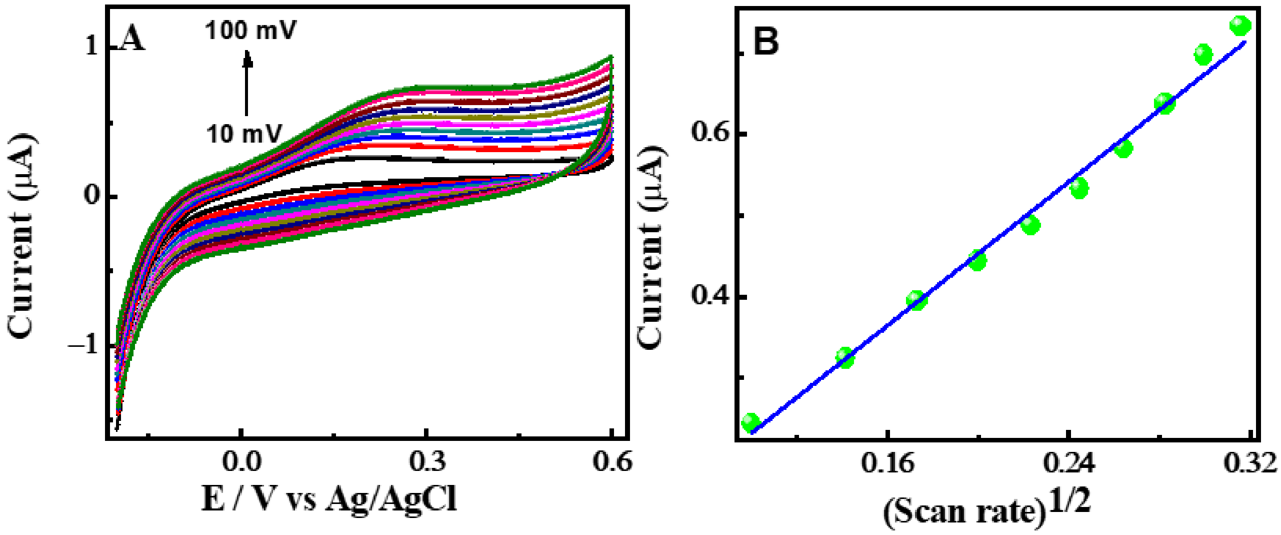

Figure S6. (**A**) Effect of varying scan rate on the CV behavior of ZnO–g–Ru–C_3N_4. (**B**) Corresponding plots of (scan rate)$^{1/2}$ versus current.

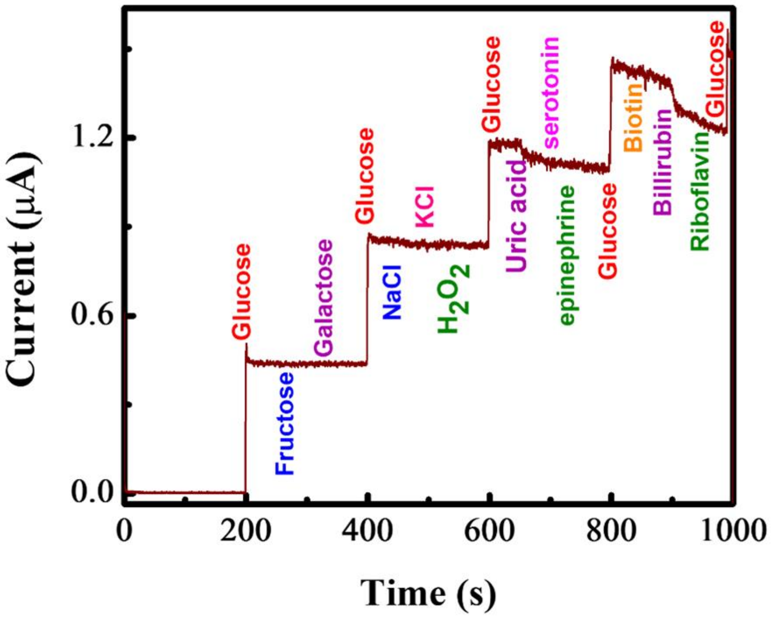

Figure S7. Selective sensing of glucose with the potential interferences are fructose, galactose, riboflavin, billurubin, biotin, NaCl, KCl, H_2O_2, uric acid, serotonin and epinephrine with the concentration of 0.1 mM in PBS.

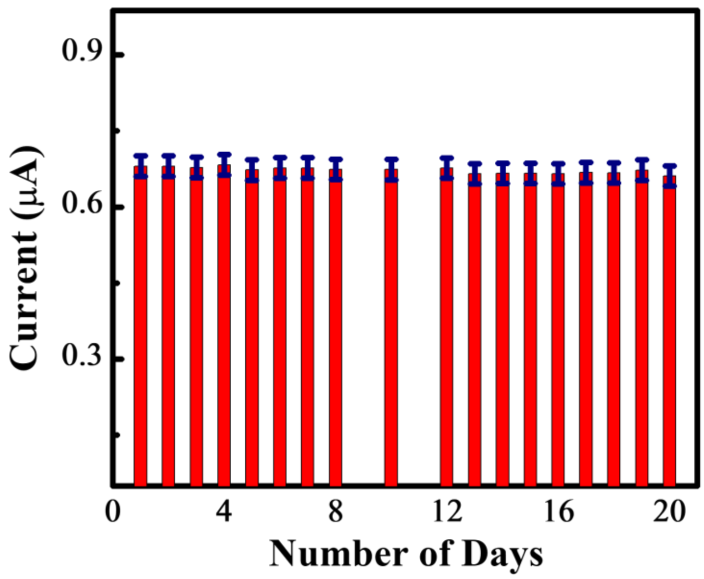

Figure S8. Study of stability of ZnO–g–Ru–C_3N_4 modified electrode monitored voltammetrically in presence of 1 mM glucose in PBS. Anodic peak current is plotted against Number of days.

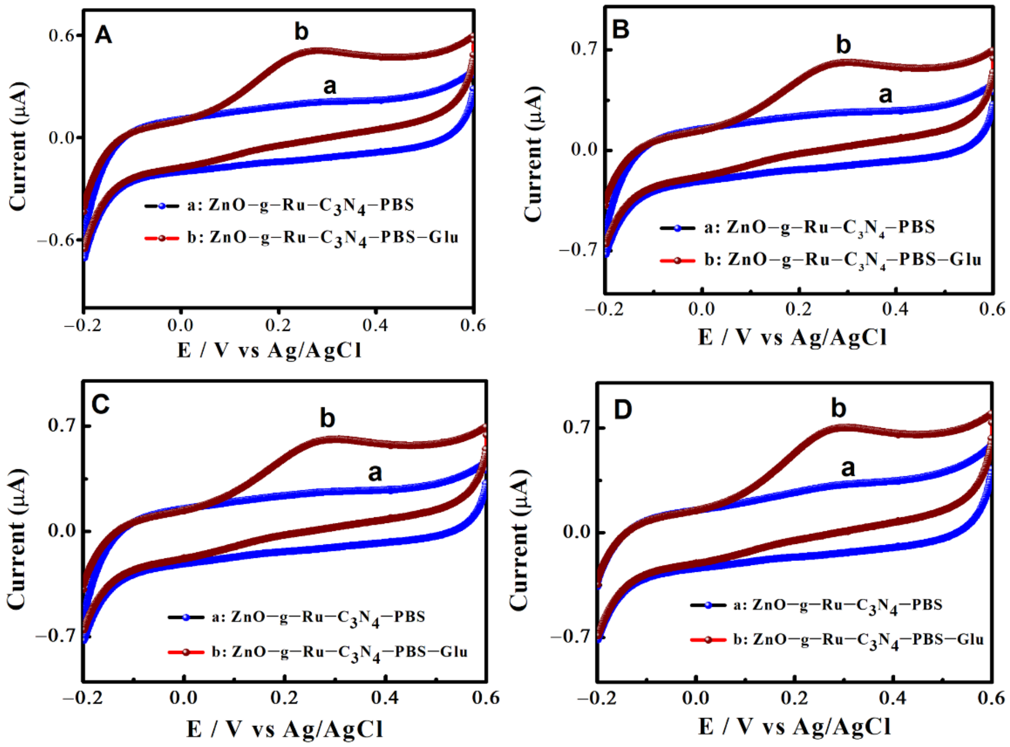

Figure S9. Reproducibility data obtained on using four different gold electrodes of similar activity for modifying with ZnO–g–Ru–C_3N_4. Measured in PBS in the absences (curve a) and presences (curve b) of 1 mM glucose at a scan rate of 50 mV s^{-1}.

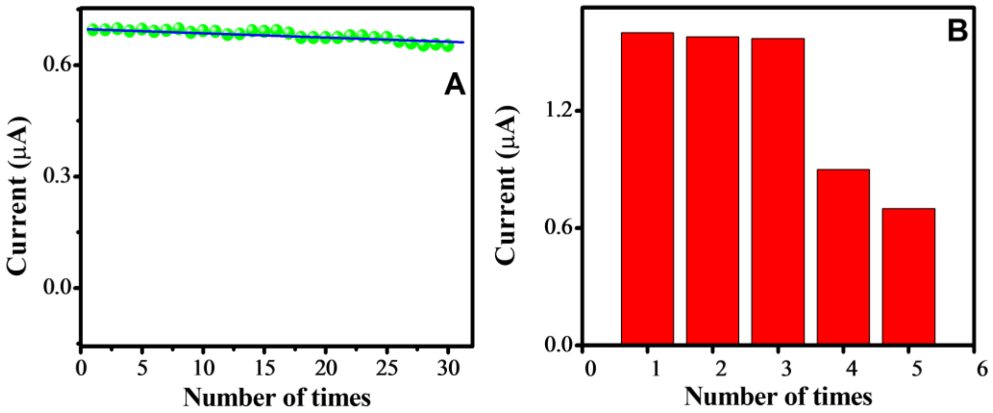

Figure S10. Reusable single electrode is measured in PBS (**A**) in the presences of 1 mM glucose at a scan rate of 50 mV s^{-1}. (**B**) Blood serum.

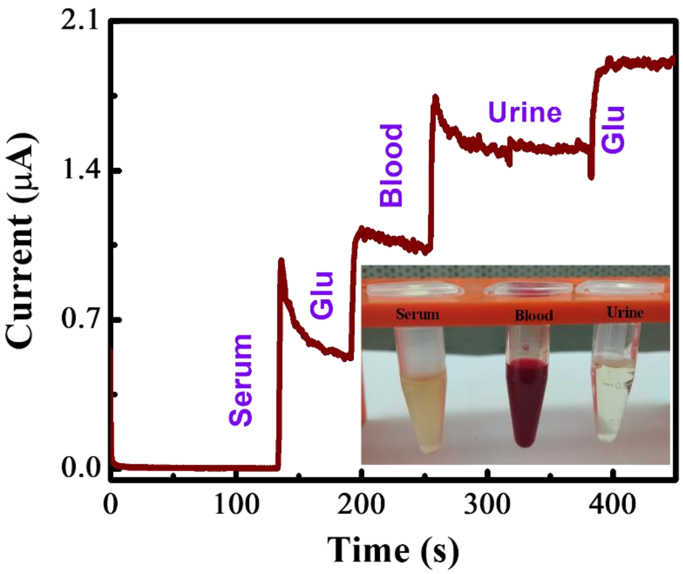

Figure S11. Developed sensor device practically tested in human blood, blood serum and urine samples.
